# Quantitative EEG as a diagnostic and prognostic tool in hemispheric stroke patients undergoing type A aortic dissection surgery

**DOI:** 10.1002/brb3.3091

**Published:** 2023-05-21

**Authors:** Ya‐peng Wang, Wen‐xue Liu, Yi Jiang, Shan Lu, Yong‐qing Cheng, Yang Chen, Dong‐jin Wang

**Affiliations:** ^1^ Department of Cardio‐Thoracic Surgery Nanjing Drum Tower Hospital, Chinese Academy of Medical Sciences & Peking Union Medical College Nanjing China; ^2^ Department of Cardiothoracic Surgery Nanjing Drum Tower Hospital, The Affiliated Hospital of Nanjing University Medical School Nanjing China; ^3^ Institute of Cardiothoracic Vascular Disease Nanjing University Nanjing China

**Keywords:** amplitude‐integrated electroencephalography, quantitative EEG, relative band power, stroke, type A aortic dissection

## Abstract

**Objective:**

The diagnostic and prognostic value of quantitative electroencephalogram (qEEG) parameters, specifically the symmetry of amplitude‐integrated electroencephalography (aEEG) and relative band power (RBP), in the postoperative stroke of the cerebral hemisphere following type A aortic dissection, remains an area of inquiry.

**Methods:**

We analyzed and processed 56 patients with type A aortic dissection who underwent bedside qEEG monitoring and analyzed the qEEG indices, brain CT, and clinical data of these patients. qEEG (symmetry of aEEG and RBP, and affected/unaffected hemisphere) indices were analyzed at discharge and 60 days after discharge.

**Results:**

A total of 56 patients were studied. The 60‐day mortality rate was 12.5%. The affected hemisphere's diagnosis and mortality after 1‐year follow‐up were evaluated, and RBP beta demonstrated the highest area under the curve values with 95% confidence intervals (CI) of .849 (95% CI: .771–.928) and .91 (95% CI: .834–.986), respectively. According to the results of the logistic regression analysis, we have identified the strongest predictors for cerebral hemisphere stroke and 1‐year mortality in stroke patients. Specifically, aEEGmin exhibited the highest predictive power with an odds ratio (OR) of .735 for cerebral hemisphere stroke, whereas DTABR was confirmed as one of the strongest predictors with an OR of 1.619 for 1‐year mortality in stroke patients, indicating a high level of reliability. Spearman correlation coefficients showed that aEEGmax and aEEGmin were positively correlated with Alberta Stroke Program Early CT Score (aEEGmax: rho = .50, *p* < .001; aEEGmin: rho = .44, *p* < .001).

**Conclusions:**

QEEG has been proven to be a sensitive indicator for monitoring brain function and can be monitored continuously. It can help clinicians detect and treat these patients early and improve long‐term prognosis.

## Graphical abstract

In our study, qEEG has been proven to be a sensitive indicator for monitoring brain function and can be monitored continuously. It can help clinicians detect and treat these patients early and improve long‐term prognosis.

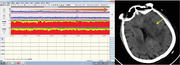

## 1 LIMITATIONS

This retrospective study presents some limitations that need to be considered. First, the sample size is limited, and there is no uniform timing for qEEG measurements among postoperative patients. Additionally, the lack of raw EEG data hinders the evaluation of stroke diagnosis and prognosis. To validate the diagnostic and prognostic effectiveness of qEEG in detecting brain injury after AAD, well‐designed and large‐scale clinical studies are required.

## 2 INTRODUCTION

Type A aortic dissection (AAD) is a grave condition that carries a mortality rate of 50% within 48 h, emphasizing the urgent need for emergency surgical intervention (Tang et al., 2021). Despite advancements in medical technology, the 30‐day postoperative mortality rate for AAD has only decreased from 30% to 12.2% (Parikh et al., 2017). Neurological complications remain a significant concern due to the concurrent low perfusion of brain tissue, extended periods of cardiopulmonary bypass (CPB), and deep hypothermic circulatory arrest (DHCA) (Centofanti et al., 2016).

Post‐stroke complications are frequently associated with perioperative brain injury. Reduced cerebral blood perfusion caused by cerebral edema or stroke can lead to diminished oxygen and glucose supply, ultimately resulting in cerebral infarction (Berger & Hakim, 1986; Bossone et al., 2013). The diagnosis of ischemic stroke relies heavily on neuroimaging and clinical assessment (Jadhav et al., 2020). Techniques such as MRI or CT perfusion can identify ischemic cores and potentially salvageable hypo‐perfused penumbras (Dashtbani Moghari et al., 2021). Unfortunately, these methods are not practical for monitoring the development of cerebral ischemia in the acute phase of patients at risk of transport after AAD surgery (Jordan, 2004). Electroencephalography (EEG) represents a noninvasive technology with high temporal resolution capable of a rapid assessment of transient brain function. It is highly sensitive to acute changes in cerebral blood flow and nerve metabolism, making it a valuable tool for bedside monitoring of brain function in emergency situations (Hellstrom‐Westas & Rosen, 2005). The subacute and post‐acute changes in EEG associated with ischemic stroke have been widely studied (Ajcevic et al., 2021).

Despite the high incidence of neurological deficits after AAD surgery, only a limited number of studies have explored early EEG changes in affected patients. The complexity and interpretation difficulty associated with traditional EEG manipulation has made it challenging for clinicians to make timely diagnoses. To address this issue, our study aims to employ quantitative electroencephalogram (qEEG) to achieve early diagnosis of neurological impairment after AAD surgery and predict patient prognosis. By leveraging the quantitative aspects of this technique, we hope to provide clinicians with a more accessible and reliable tool for assessing the neurological status of patients after AAD surgery.

## 3 MATERIALS AND METHODS

### 3.1 Study population and protocol

Between October 2021 and April 2022, the Department of Cardiothoracic Surgery at Nanjing Drum Tower Hospital treated 136 patients with AAD. Inclusion criteria for this study involved performing bedside qEEG monitoring after AAD surgery. Exclusion criteria included patients who received sedatives or were sedated within 1 h prior to monitoring, patients with obvious sequelae such as large area cerebral hemorrhage or cerebral infarction, and patients with a history of epilepsy, craniotomy for brain tumor, or craniocerebral trauma. The Medical Ethics Committee of Nanjing Drum Tower Hospital approved the study protocol, and the need for informed consent was waived as patient records and information were anonymized and de‐identified before analysis.

### 3.2 qEEG acquisition and processing

This was a retrospective study that involved bedside qEEG monitoring of patients using a Nicolet Monitor brain function instrument (NicoletOne 5.9.4, Natus Neurology Incorporated). Each patient was monitored at least once for a minimum of 20 min. The study analyzed two parameters of qEEG, namely, the amplitude‐integrated EEG (aEEG) and relative band power (RBP), which were used to assess and quantify changes in brain function after AAD surgery.

The international 10–20 system was used to place electrodes for qEEG monitoring, and data was recorded through the longitudinal connection of F3–P3 and F4–P4. A filter of 1–35 Hz was applied during data collection. The aEEG data was automatically extracted every 1 s and displayed in a semilogarithmic compression format to reflect changes in EEG signal amplitude. The upper and lower boundaries of aEEG were automatically extracted by the computer. RBP was calculated every 10 s, and the percentages of delta (0.5–4 Hz), theta (4–8 Hz), alpha (8–13 Hz), and beta (13–30 Hz) were calculated and color‐coded (Figure 2). Overall, these techniques were used to assess the changes in brain function in response to AAD surgery.

To evaluate the usefulness of qEEG in detecting cerebral infarction in AAD, we extracted all spectral parameters and exported them to Excel files. These files were used for further analysis to assess the differences in qEEG between the left and right brain in patients with cerebral infarction following AAD surgery.

## 4 STATISTICAL ANALYSES

In this study, variables were reported as either mean and standard deviation or median and range, depending on the distribution. The normal distribution of variables was evaluated using the Kolmogorov–Smirnov test. Categorical variables were compared using the Chi‐square test. The relationship between the Alberta Stroke Program Early CT Score (ASPECTS) and qEEG parameters was assessed individually using Spearman correlation analysis. A statistically significant result was defined as a *p*‐value <.05. To identify the most relevant independent qEEG indices related to post‐stroke outcomes, stepwise multiple regression analysis was conducted. The data analysis was performed using R software version 4.2.2.

## 5 RESULTS

**FIGURE 1:**
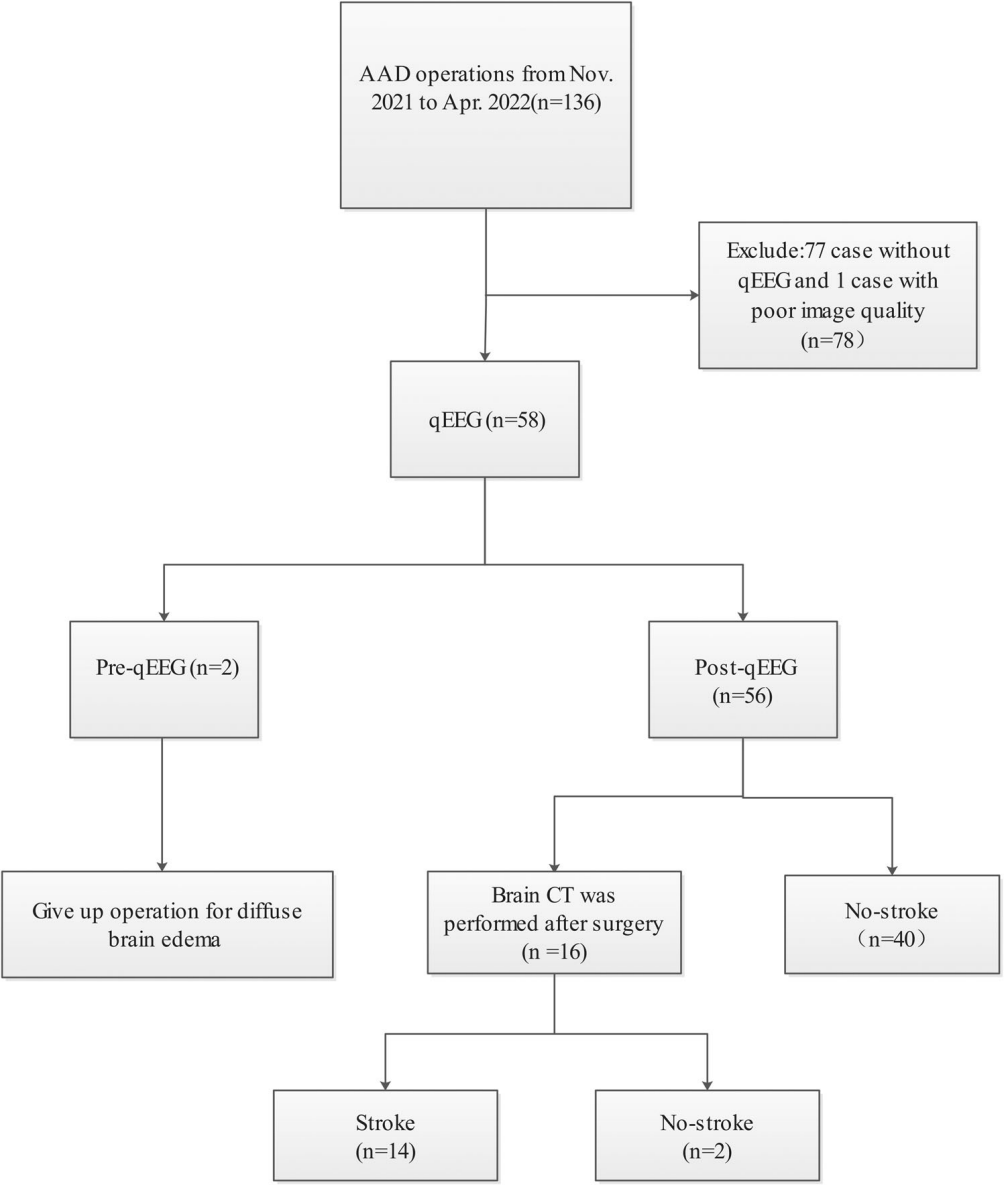
Flow chart of study.

Between November 2021 and April 2022, a total of 136 patients with AAD underwent aortic repair procedures. Among them, 80 patients were excluded from the study, including 77 who did not undergo qEEG, 2 who opted out of the operation due to diffuse brain edema, and 1 with poor qEEG image quality. Ultimately, 56 subjects were enrolled in this study, as shown in Figure 1. A total of 112 qEEG analyses were performed, with 56 conducted on the left cerebral hemisphere and 56 on the right.

**FIGURE 2:**
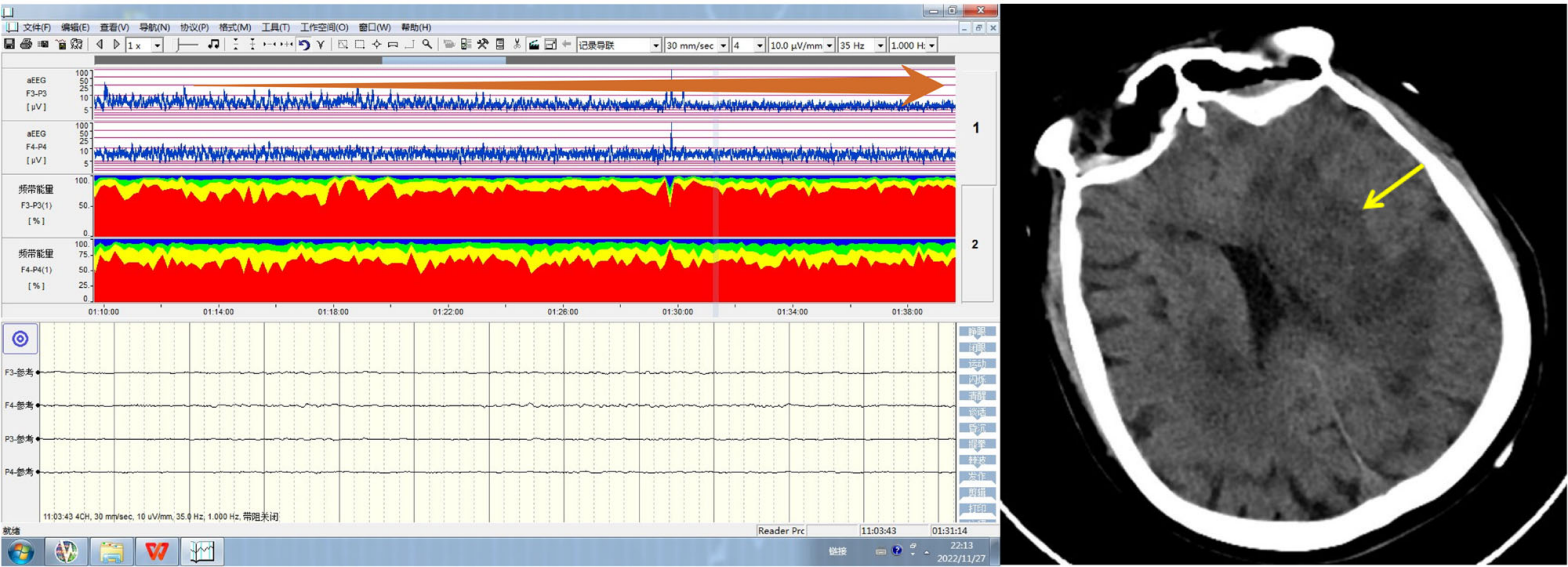
Asymmetry of amplitude‐integrated electroencephalography (aEEG) in the stroke patients. The red arrow indicated that aEEG of F3–P3 started to decrease on the left cerebral hemisphere compared with aEEG on the right side, and the yellow arrow indicated large cerebral infarction on the left side.

Table 1 displays the baseline characteristics of the two groups, which showed no significant differences in age, BMI, sex, smoking, alcohol consumption, diabetes, hypertension, chronic kidney disease, chronic obstructive pulmonary disease, immune system disease, and coronary heart disease (*p* > .05). Additionally, there were no significant differences in the surgical repair of aortic arch, left common carotid artery stent, right common carotid artery stent, innominate artery stent implantation, coronary artery bypass, pulmonary thrombectomy, heart valve surgery, and surgical status between the two groups (*p* > .05). The prestenting operation time, CPB, cross‐clamp, ventilation time, CCU stay, and 60‐day mortality data were comparable between the two groups (*p* < .05), except for cross‐clamp, DHCA, and hospital stay.

**TABLE 1:** Clinical demographics for no‐stroke and stroke

Demographics	No‐stroke (*n* = 42)	Stroke (*n* = 14)	*p*
Age (years)	58(49.5, 68.3)	66.5 (50.5, 71.3)	.25
Body mass index	24.5 ± 3.6	26.6 ± 2.5	.08
Male sex	31	10	.64
Smoking	8	5	.36
Alcohol	5	2	.57
Diabetes	2	0	.10
Hypertension	35	13	.67
Chronic kidney disease	5	0	.31
Chronic obstructive pulmonary disease	1	0	1.00
Coronary heart disease	3	1	1.00
Immune system disease	4	0	.56
Stroke	1	2	.15
Surgical approach to aortic arch			.907
Subtotal arch replacement	5	4	
Island anastomosis	22	7	
Fenestrated arch stent	9	4	
Total aortic arch replacement	3	2	
Left common carotid artery stent	3	2	.59
Innominate artery stent	7	1	.66
Right common carotid artery stent	0	1	.25
Coronary artery bypass	1	1	.44
Pulmonary thrombectomy	1	0	1.00
Heart valve surgery	4	2	.64
Surgical status			.869
Elective	7	6	
Urgent	21	7	
Salvage	11	4	
Operation time (min)	388.3 ± 89.6	468.2 ± 121.3	.011
CPB (min)	168.0 (152.0, 196.3)	202.0 (161.3, 240.8)	.042
Cross‐clamp (min)	127.0 (110.5, 145.3)	141.0 (115.5, 141.0)	.24
DHCA (min)	24.0 (18.8, 145.3)	21.0 (18.0, 32.0)	.93
Ventilation time (h)	16.0 (10.8, 62.7)	197.2 (88.5, 324.7)	.001
CCU stay (day)	4 (2, 7)	10.5 (6.25, 16.5)	.002
Hospital stay (day)	13 (10.5, 19.0)	15.0 (8.5, 30.0)	.90
60‐day mortality	1	6	.001

**FIGURE 3:**
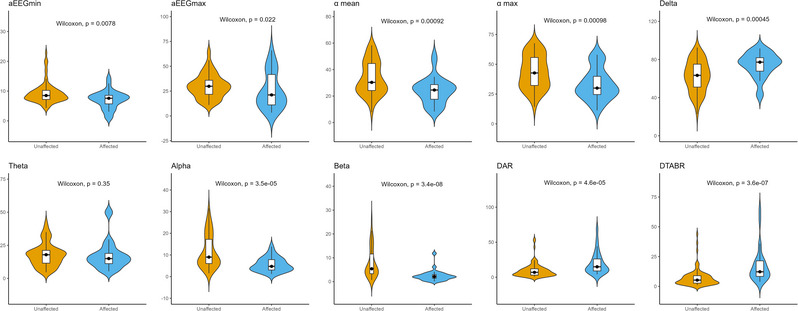
Violin plots of the quantitative electroencephalogram (qEEG) indices between unaffected and affected groups.

**FIGURE 4:**
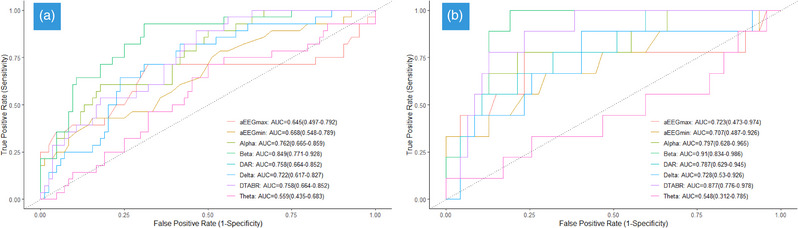
Comparison of receiver operating characteristic (ROC) curves to predict the stroke (A) and death (B) after 1‐year follow‐up between the quantitative electroencephalogram (qEEG) indices in this cohort.

Table 2 shows that, besides theta waves, other qEEG parameters exhibited significant differences between the affected and unaffected groups. Violin plots of the qEEG indices for the affected and unaffected groups are presented in Figure 3. Table 3 presents the results in terms of the presence of stroke survivor and non‐survivor groups after 1‐year follow‐up. In addition to aEEGmin and RBP theta, statistical differences were observed in all other qEEG parameters. As Figure 4A,B shows, the highest area under the receiver operating characteristic of the RBP beta for stoke and death was 0.849 (95% confidence intervals [CI]: .771–.928) and .910 (95% CI: .834–.986), respectively.

**TABLE 2:** Quantitative electroencephalogram (qEEG) indices between groups of unaffected and affected cerebral hemisphere

qEEG index	Unaffected (*n* = 84)	Affected (*n* = 28)	*p* (2‐tailed)
aEEGmax	29.9 (21.8, 37.4)	20.4 (10, 41.7)	.007
aEEGmin	8.5 (7.2, 10.2)	7.4 (4.7, 8.6)	.002
Delta	63.6 (51.1, 76.0)	77.6 (67.8, 86.7)	.000
Theta	17.6 (10.8, 21.2)	14.0 (9.9, 14.0)	.217
Alpha	8.8 (5.9, 17.2)	4.4 (2.8, 7.8)	.000
Beta	5.4 (3.0, 11.6)	2.1 (1.1, 3.3)	.000
DAR	7.1 (3.1, 13.0)	15.3 (9.0, 31.3)	.000
DTABR	5.4 (2.7, 9.0)	13.4 (8.4, 21.7)	.000

Abbreviations: DAR, delta/alpha power ratio; DTABR, (delta + theta)/(alpha + beta) power ratio.

**TABLE 3:** Quantitative electroencephalogram (qEEG) indices between groups of stroke survivors and non‐survivors after 1‐year follow‐up

qEEG index	Survivor (*n* = 47)	Death (*n* = 9)	*p* (2‐tailed)
aEEGmax	28.6 ± 12.6	19.7 ± 16.9	.07
aEEGmin	8.1 (6.8, 10.2)	6.6 (3.1, 8.5)	.05
Delta	64.1 ± 15.6	76.0 ± 15.0	.04
Theta	17.3 (10.9, 20.2)	14.5 (7.9, 21.4)	.65
Alpha	8.7 (5.5, 16.1)	3.8 (2.8, 7.2)	.005
Beta	5.0 (3.0, 11.4)	1.8 (1.0, 2.2)	.000
DAR	7.9 (3.7, 13.0)	23.0 (9.1, 30.4)	.007
DTABR	6.2 (2.8, 9.5)	15.1 (10.9, 21.2)	.000

Abbreviations: DAR, delta/alpha power ratio; DTABR, (delta + theta)/(alpha + beta) power ratio.

Table 4 displays the results of logistic regression analysis conducted on aEEGmax, aEEGmin, DAR, and DTABR, in order to predict the affected cerebral hemisphere. The findings demonstrate that aEEGmin emerges as the most robust and independent predictor, with an odds ratio (OR) of .735. The statistical findings revealed that DTABR was the strongest and independent predictor of the death after 1‐year follow‐up, with an OR value of 1.619 as shown in Table 4.

**TABLE 4:** Logistic regression analysis of the most relevant independent quantitative electroencephalogram (qEEG) indices for postoperative stroke survivors and non‐survivors after 1‐year follow‐up

qEEG index	Terminal event	β	OR	95% CI	
				Lower limit	Upper limit
aEEGmax	Stroke	.014	1.015	.957	1.075
aEEGmin	Stroke	−.308	.735	.543	0.996
DAR	Stroke	−.052	.950	.871	1.036
DTABR	Stroke	.118	1.125	.986	1.285
aEEGmax	Death	−.106	.900	.792	1.021
aEEGmin	Death	.111	1.117	.681	1.833
DAR	Death	−.265	.767	.565	1.041
DTABR	Death	.482	1.619	1.044	2.513

Abbreviations: DAR, delta/alpha power ratio; DTABR, (delta + theta)/(alpha + beta) power ratio; OR, odds ratio.

**FIGURE 5:**
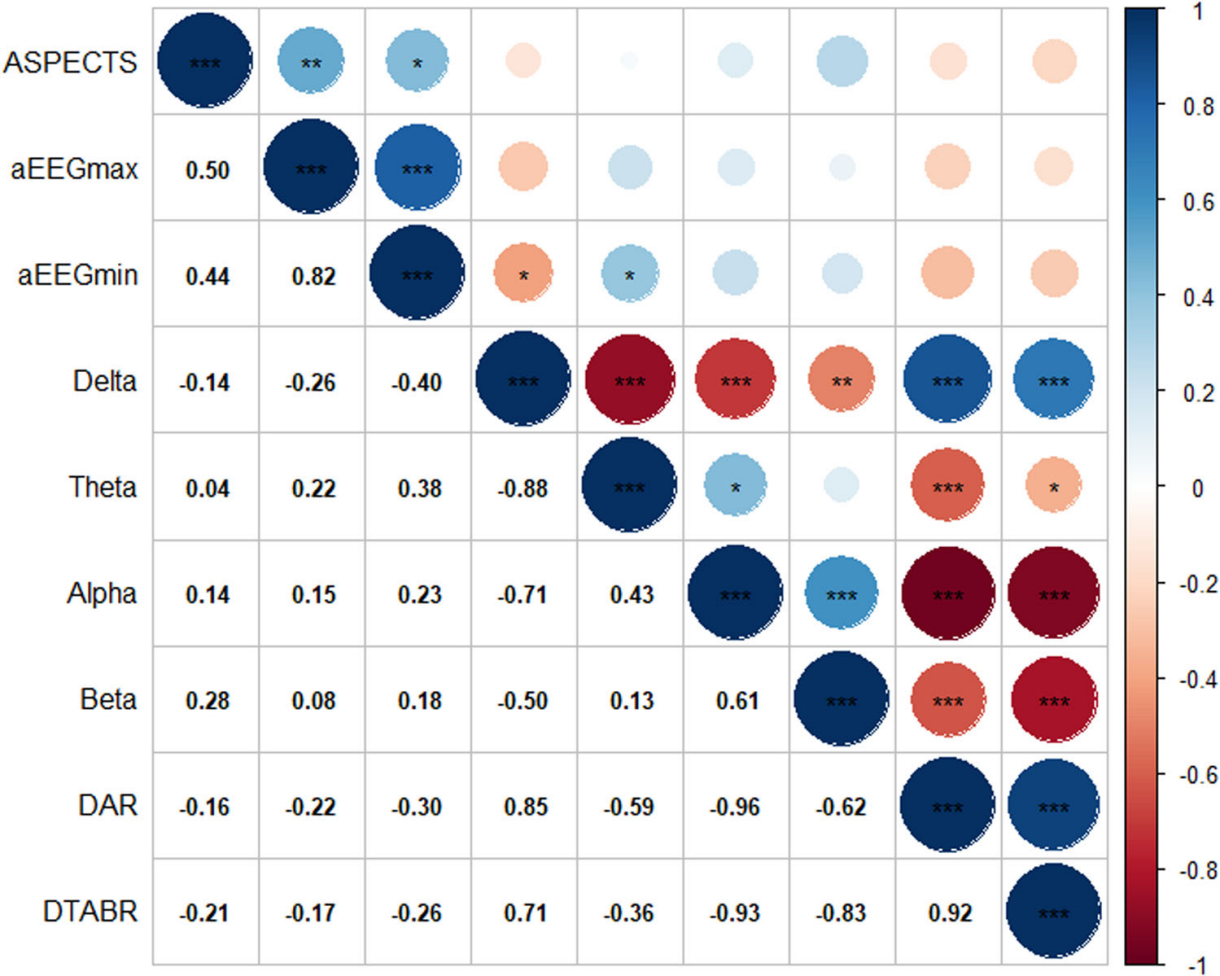
Matrix of Spearman's correlation coefficients between Alberta Stroke Program Early CT Score (ASPECTS) and nine quantitative electroencephalogram (qEEG) indices. A color‐coded correlation scale is presented on the right of the plot. Based upon the scale, red ones stand for negative correlations and blue ellipses stand for positive correlations. Crossed out boxes illustrate insignificant correlations of a given variable with itself. Red star symbols represent statistical significance levels: “***” represents *p* < .001, “**” represents *p* < .01, “*” represents *p* < .05, no stars represent *p* > .05.

Table 5 displays that aEEGmax was significantly different between stroke patients with ASPECTS (≤5) and ASPECTS (≥6). Figure 5 presents Spearman correlation coefficients, indicating that aEEGmax and aEEGmin were positively correlated with ASPECTS (aEEGmax: rho = .50, *p* < .001; aEEGmin: rho = .44, *p* < .05).

**TABLE 5:** Comparison between Alberta Stroke Program Early CT Score (ASPECTS) (≥6) and ASPECTS (≤5) of quantitative electroencephalogram (qEEG) indices characteristics

qEEG index	ASPECTS (≥6) (*n* = 22)	ASPECTS (≤5) (*n* = 10)	*p* (2‐tailed)
aEEGmax	24.3 (13.9, 45.7)	13.0 (6.4, 20.8)	.004
aEEGmin	8.1 (6.5, 9.2)	6.5 (2.6, 8.0)	.051
Delta	76.9 (65.6, 80.4)	81.5 (74.5, 89.0)	.088
Theta	13.6 (11.3, 21.2)	14.0 (6.1, 17.4)	.272
Alpha	5.0 (3.7, 7.8)	3.4 (2.4, 6.0)	.113
Beta	2.2 (1.4, 3.9)	1.8 (0.4, 2.7)	.180
DAR	12.4 (9.0, 22.1)	25.0 (13.2, 38.8)	.067
DTABR	10.8 (7.4, 18.9)	17.5 (11.4, 30.3)	.092

Abbreviations: DAR, delta/alpha power ratio; DTABR, (delta + theta)/(alpha + beta) power ratio.

**FIGURE 6:**
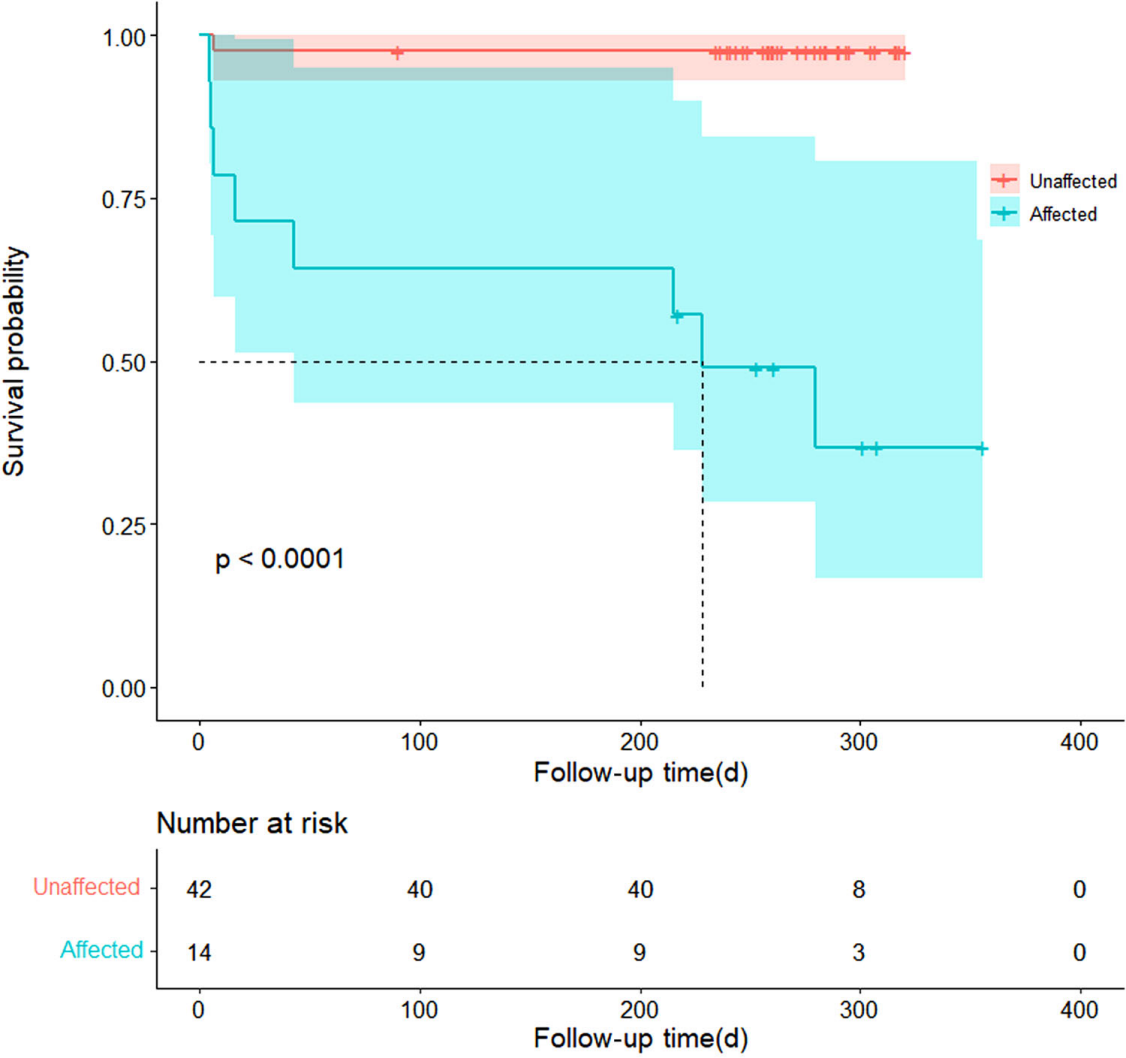
Kaplan–Meier survival curve for stroke and non‐stroke patients.

After 1‐year follow‐up, nine (16.1%) patients had died. The Kaplan–Meier survival curve showed a trend toward poorer survival rates among patients without stroke (*p* < .001), as demonstrated in Figure 6.

## 6 DISCUSSION

The changes in brain activity during postoperative neurological impairment in AAD are believed to be linked to neurophysiological alterations that occur when brain tissue perfusion is inadequate (Rossini et al., 2004). This mechanism is known as neurovascular coupling (Stragapede et al., 2019). In particular, the decrease in CBF in the ischemic area results in changes in EEG activity, mainly an increase in delta frequency power and a reduction in alpha frequency power (Jordan, 2004). Extensive research has been conducted on EEG changes in the subacute and acute phases of ischemic stroke (Ajcevic et al., 2021; Finnigan et al., 2004, 2007; Sheorajpanday et al., 2011; Wu et al., 2016).

The transition from ischemia to infarction in the acute stage of ischemic stroke is a rapid process, typically taking only a few minutes to several hours from the onset of hypoperfusion to cell death (Murri et al., 1998). EEG has been shown to be a sensitive indicator of brain injury and can detect early changes in neurological function (Ajcevic et al., 2021). Previous studies (Finnigan & van Putten, 2013; Finnigan et al., 2007) have demonstrated that qEEG technology is capable of quantifying the amplitude, frequency, and spatial distribution of electrical activity in the cerebral cortex.

Early detection of brain complications after surgery for AAD is critical to improve long‐term prognosis (Bae et al., 2005). However, due to factors such as invasive mechanical ventilation and delayed awakening, patients may not be able to undergo an effective physical examination of the nervous system. Additionally, transporting patients for CT or MRI scans of the brain can increase risks and is not always feasible in a clinical setting (Smith‐Bindman, 2010; Vilela & Rowley, 2017). Therefore, alternative methods for early detection, such as qEEG, may be valuable for these patients.

Although continuous low voltage and electrocerebral silence on aEEG have been previously linked to poor prognosis, the use of left–right EEG activity asymmetry as a diagnostic and prognostic indicator for acute stroke has not been widely studied (Rundgren et al., 2006; Tao & Mathur, 2010; ter Horst et al., 2004). Our study shows that reduced EEG activity on the affected side results in bilateral EEG asymmetry, which can predict the extent of brain injury. The greater the asymmetry, the more severe the patient's condition, and the worse the long‐term neurological prognosis. Additionally, RBP in qEEG uses color to indicate the proportion of δ, θ, α, and β in the trend map. In ischemic and hypoxic conditions, the proportion of δ waves increases in the affected brain, leading to noticeable asymmetry in both sides of the brain. This RBP‐derived asymmetry may further aid in the diagnosis of acute stroke.

In this study, we investigated the prognostic value of several qEEG indices, including aEEGmin, slow wave fraction, and RBP symmetry measures, in predicting poor neurological outcome. Our findings demonstrated that these qEEG indices were all independent predictors of poor neurological outcome, which is consistent with previous studies (Bentes et al., 2018; Sandroni et al., 2020; Scheuer & Wilson, 2004). Additionally, we observed a positive linear correlation between aEEG and ASPECTS, with aEEG being the strongest predictor of affected cerebral hemisphere. These qEEG indices are similar to the results of background asymmetric visual EEG analysis reported in previous studies (de Vos et al., 2008).

The Modified Rankin Scale and the Barthel Index are widely used tools to assess functional disability and dependence in patients with neurological conditions. However, due to missing data, these measures were not included in our retrospective study. In contrast, the ASPECTS scoring system is a widely recognized and reliable tool for assessing the severity of ischemic stroke (Menon et al., 2011; Pexman et al., 2001). This system requires only CT scan and has been extensively validated, providing crucial information about patient conditions and prognosis (Naylor et al., 2017). Our study revealed a linear correlation between aEEG and ASPECTS, suggesting that aEEG may serve as an important predictor of neurological prognosis. In particular, we found that aEEGmax and aEEGmin were linearly correlated with ASPECTS, underscoring the importance of aEEG as a crucial indicator of neurological outcomes. Overall, our findings highlight the value of accessible and effective diagnostic tools in the management of ischemic stroke patients.

Studies (Colombo et al., 2019; Lanzone et al., 2022) have shown that spectral exponent (SE), an index reflecting EEG slowing and quantifying power spectral density with power law decay, can be a valuable metric for assessing the neural physiological state of cortical circuits after focal ischemic injury. Comparing SE between the affected and unaffected hemispheres can aid in guiding neurorehabilitation efforts (Donoghue et al., 2020). Unfortunately, our qEEG equipment can only monitor SE for the entire brain and was therefore unable to be included in this study.

Previous research studies have suggested a negative correlation between alpha activity and stroke prognosis (Diedler et al., 2010; Schleiger et al., 2014). Additionally, studies of EEG activity in patients undergoing carotid endarterectomy have shown that alpha changes occur earlier than slow waves (Sharbrough et al., 1973). However, in the case of aortic type A dissection, DHCA is required, which results in the whole brain EEG being in a state of electrical resting inhibition, and bilateral brain RBP being mainly theta waves. During the awakening process in the intensive care unit, the changes in the overall pattern and proportion of theta waves in both sides of the brain were most prominent. This difference in findings from other studies may be attributed to these factors.

Our study revealed that patients with cerebral infarction had a lower 60‐day survival rate compared to those without cerebral infarction, and this difference was statistically significant. Moreover, after 1‐year of follow‐up, the difference in survival rates remained significant, as shown in Figure 5. In contrast, the study has demonstrated that cerebral infarction increased the hospitalization rate of patients without affecting their long‐term mortality (Bossone et al., 2013). However, our results may be attributed to the larger size of infarcts in our patient population, as well as lower medical compliance after discharge.

## 7 CONCLUSION

In summary, our study investigated the association between early EEG changes and neurological impairment in patients with ischemic stroke after aortic dissection. Our findings indicate that qEEG is a sensitive tool for monitoring brain function, and its continuous monitoring can aid clinicians in the early detection and treatment of brain complications, leading to improved long‐term prognosis.

## AUTHOR CONTRIBUTIONS

Ya‐peng Wang, Wen‐xue Liu, Yi Jiang, Shan Lu, Yang Chen, and Dong‐jin Wang contributed to study concept and design; Ya‐peng Wangand Wen‐xue Liu involved in acquisition of data; Yang Chen and Dong‐jin Wang contributed to drafting the manuscript; all authors read and approved the final manuscript. All authors have confirmed that manuscript complies with all instructions to us. All of authors confirmed that this manuscript has not been published elsewhere and is not under consideration by another journal.

## CONFLICT OF INTEREST STATEMENT

All authors declare that they have no conflict of interests.

### PEER REVIEW

The peer review history for this article is available at https://publons.com/publon/10.1002/brb3.3091.

## Data Availability

The data used to support the findings of this study are available from the corresponding author upon reasonable request.
